# Use of Ox Gall in Disease

**Published:** 1860-08

**Authors:** B. Woodward

**Affiliations:** Galesburg, Ill.


### ON THE USE OF OX GALL IN DISEASE.

BY B. WOODWARD M. D., Galesburg, Ill.

The physiological action of the bile has been thoroughly studied; to some extent its action is understood. Its therapeutical action has also been partially investigated; yet to very many of the profession it is still almost unknown as a remedial agent. The chemical analogy existing between some of its constituents and some of the vegetable alkaloids, particularly quinia, has been pointed out by Liebig and others, and its use in some forms of dyspepsia has been recommended. Having to some extent made use of it—I beg leave to occupy your columns with a few lines on the subject.

We not unfrequently find forms of both remittent and intermittent fevers, in which the bowels are constipated, the face clay colored, the appetite either entirely wanting, or depraved; the spleen enlarged, the urine scanty, turbid or ammoniacal; the excrementitious matters which should have been eliminated by the kidneys retained, and a state of *uremic poisoning*, and what, for want of a better term, I would call *Hydro-carbonic poisoning;* evidenced by a sallow anæmic countenance, pain in the regions of the liver and kidneys, and such a deparation of the blood that its red corpuscles are notably diminished. All the symptoms point to an arrest of the hepatic functions, and in which mercurials are indicated to rouse the liver from its torpor, and stimulate the intestinal circulation.

But we need also some agent which shall give tone to digestion and assimilation, when once the hepatic functions have been arrested, or seriously deranged, it requires time as well as remedials to restore them, and in the meantime the system requires some agent to take the place of the natural secretions of the liver to carry on digestion, assimilation and depuration. Inspissated ox gall will be found to answer the demands of the system. Not only is the bile excrementitious, but a large, probably the largest part, is re-absorbed, after having undergone a chemical change in the intestines, and carried into the circulation to aid in assimilation and depuration. Recent physiological investigations have evidenced that the liver is a sugar elaborating organ. This sugar being in the form of glycocine or gelatine sugar, cannot be supplied to the system in any other way; and the want of it in torpid states of the liver may be one cause of the long train of morbid symptoms growing out of this condition. This sugar exists in the bile of all animals, and may be supplied to the system by the use of the gall of the ox, till such times as the liver has regained its healthy action. By the use of inspissated ox gall, we obtain all the constituents of bile, except the mucus, which from its nature must be wholly excrementitious.

These are some of the reasons which have led me to the use of ox gall in the treatment of disease. I would not claim that it will arrest a paroxysm of intermittent, but its use in conjunction with quinia has been eminently beneficial in restoring digestion and assimilation, and also in restoring the depurative action of the kidneys. Combined with iron by hydrogen in the form of pill, it will be found a valuable restorative after intermittents and remittents, keeping the bowels soluble, and stimulating digestion, while the iron will have its own restorative action. In jaundice, from want of biliary secretion, it will also be found a valuable agent, as well as in those forms of dyspepsia depending on hepatic derangement. From 3 to 8 grains, twice a day, is as much as will generally be found necessary. I will only add, that as a laxative in chronic constipation of the bowels, its use combined with bi-carb. soda has in my hands been of marked benefit.

Among your correspondents there are doubtless those who have examined this subject with care; and if they would give us the results of their observations, they would benefit us by throwing more light upon it.

